# The Induction of microRNA-16 in Colon Cancer Cells by Protein Arginine Deiminase Inhibition Causes a p53-Dependent Cell Cycle Arrest

**DOI:** 10.1371/journal.pone.0053791

**Published:** 2013-01-07

**Authors:** Xiangli Cui, Erin E. Witalison, Alena P. Chumanevich, Alexander A. Chumanevich, Deepak Poudyal, Venkataraman Subramanian, Aaron J. Schetter, Curtis C. Harris, Paul R. Thompson, Lorne J. Hofseth

**Affiliations:** 1 Department of Drug Discovery and Biomedical Sciences, South Carolina College of Pharmacy, University of South Carolina, Columbia, South Carolina, United States of America; 2 Shanxi Medical University, Shanxi, China; 3 Department of Chemistry, The Scripps Research Institute, Scripps Florida, Jupiter, Florida, United States of America; 4 Laboratory of Human Carcinogenesis, Center for Cancer Research, National Cancer Institute, National Institutes of Health, Bethesda, Maryland, United States of America; CNRS-University of Toulouse, France

## Abstract

Protein Arginine Deiminases (PADs) catalyze the post-translational conversion of peptidyl-Arginine to peptidyl-Citrulline in a calcium-dependent, irreversible reaction. Evidence is emerging that PADs play a role in carcinogenesis. To determine the cancer-associated functional implications of PADs, we designed a small molecule PAD inhibitor (called Chor-amidine or Cl-amidine), and tested the impact of this drug on the cell cycle. Data derived from experiments in colon cancer cells indicate that Cl-amidine causes a G1 arrest, and that this was p53-dependent. In a separate set of experiments, we found that Cl-amidine caused a significant increase in microRNA-16 (miRNA-16), and that this increase was also p53-dependent. Because miRNA-16 is a putative tumor suppressor miRNA, and others have found that miRNA-16 suppresses proliferation, we hypothesized that the p53-dependent G1 arrest associated with PAD inhibition was, in turn, dependent on miRNA-16 expression. Results are consistent with this hypothesis. As well, we found the G1 arrest is at least in part due to the ability of Cl-amidine-mediated expression of miRNA-16 to suppress its' G1-associated targets: cyclins D1, D2, D3, E1, and cdk6. Our study sheds light into the mechanisms by which PAD inhibition can protect against or treat colon cancer.

## Introduction

Dysregulated protein arginine deiminase (PAD) activity has been proposed to play a role in the onset and progression of numerous human diseases, including cancer [1]. PADs are a family of enzymes that convert peptidyl-Arginine to peptidyl-Citrulline (Arg → Cit) [2], a process called ‘citrullination’. Mammals encode 5 isozymes within a single evolutionarily conserved gene cluster, which in humans is located on chromosome 1 (1p35-36) [3]. The mammalian PAD family members, which have been designated as PADs 1–4 and PAD6, are highly related enzymes both within and between individual species. Elevated levels of PAD enzymes and/or citrullinated proteins are found in multiple chronic conditions, including colitis and colon cancer [1], [4], [5], [6], [7]. In this light, we have developed inhibitors of PADs, and one particular inhibitor, called Chlor-amidine (Cl-amidine), has been shown to have cytotoxicity against cancer cell lines [8], and can be used to prevent and treat the high colon cancer risk disease, ulcerative colitis in mice [4].

MicroRNAs (miRNA) are evolutionarily conserved, 20- to 25-nucleotide-long, noncoding RNAs that bind to their targets through partial complementary sequence recognition. Such binding results in either mRNA degradation or inhibition of translation, and therefore suppressed expression of miRNA targets [9]. Vertebrate genomes are predicted to encode as many as 1000 unique miRNAs [10], which are thought to regulate the expression of at least 30% of genes [11]. Therefore it is not surprising that miRNAs have diverse biological activities, including the regulation of cell proliferation, differentiation, and apoptosis. To this end, miRNA-16 is a putative tumor suppressor miRNA, and is down-regulated in a variety of human cancers, including colon cancer [12], [13], [14], [15], [16], [17], [18]. One recognized function of miRNA-16 is that it controls the cell cycle primarily through a G1 cell cycle checkpoint [13], [19], [20], [21], [22], [23], [24], [25], [26]. Therefore, in the absence of miRNA-16, cancer cells can grow uncontrollably. With this in mind, it has recently been demonstrated that inhibition of PADs by Cl-amidine causes induction of p21^WAF1^, leading to a G1 cell cycle arrest in colon cancer cells [27]. Building off these results, to better understand the mechanisms by which Cl-amidine causes a G1 cell cycle arrest, we show here that in the presence of p53, the G1 arrest caused by Cl-amidine is dependent on miRNA-16.

## Methods

### Cl-amidine

The synthesis of the Cl-amidine has been described previously [28], [29]. Stock concentrations of Cl-amidine were diluted in 1x Phosphate Buffered Saline (PBS) immediately before addition to cell culture.

### Cell culture

HCT 116 wild-type (WT) colon cancer cells were purchased from American Type Culture Collection (ATCC; Manassas, VA). HCT 116 p53^−/−^ colon carcinoma cells were derived from HCT 116 WT cells, and provided by B. Vogelstein and K. Kinzler. HCT 116 WT and p53^−/−^ cells were cultured in McCoy's medium (ATCC, Manassas, VA) supplemented with 10% Newborn Calf Serum (NBCS; GIBCO/Life Technologies, Grand Island, NY), 2 mM glutamine (Biofluids, Rockville, MD), penicillin (10 U/ml, Biofluids) and streptomycin (10 μg/ml, Biofluids). LS-180 colon cancer cells, purchased from ATCC, were cultured in Dulbecco's Modified Eagles Medium (Hyclone, Logan, Utah) supplemented with 10% Newborn Calf Serum (NBCS); GIBCO/Life Technologies), 2 mM glutamine (Biofluids, Rockville, MD), penicillin (10 U/mL, Biofluids) and streptomycin (10 μg/mL, Biofluids).

### siRNA and miRNA Transfection

For PAD4 siRNA, 3×10^5^ cells were grown in medium in 6 well plates 1 day before transfection. Using INTERFERin siRNA Transfection Regent (Plyplus, iLllkirch, France), the cells were transfected with 20 nmol/L of scambled siRNA as a negative control or PAD4 Trilencer-27 human siRNA (Origene, Rockville, MD). After 24 h of transfection, the cells were processed for western blot analysis to evaluate the efficacy of knock down. Cell cycle was examined after 24 h of transfection. For anti-miR-16 and miR-16-mimic (Ambion, Austin, TX) transfection, 3×10^5^ cells were grown in medium in 6 well plates 1 day before transfection. Using INTERFERin siRNA Transfection Reagent, the cells were transfected with 45 nmol/L of scrambled siRNA as negative control and anti-miR-16 or hsa-miR-16. After 24 h of transfection, the cells were harvested in RNase free EP tubes. Total RNA was extracted using TRIzol regent. RNA concentration was determined by Nanodrop 2000. 10 ng of total RNA was reverse-transcribed to cDNA using TaqMan MicroRNA Reverse Transcription kit with microRNA primers specific for hsa-miR-16 and the small nuclear protein RNU6B (U6) for normalization. qPCR measurement of miRNA-16 and U6 expression was performed using TaqMan MicroRNA Assays with the 7300 PCR Assay System. The relative fold change in miRNA-16 level was used to represent the relative abundance of miRNA-16 compared with U6 expression. After 24 h of transfection with anti-miR-16, Cl-amidine (50 µg/mL) was added and harvested for cell cycle analysis at 0 h, 2 h, 8 h, and 24 h. Cell cycle was also examined 24 h after transfection of the miRNA-16 mimic. All experimental control samples were treated with an equal concentration of a non-targeting control mimic sequence or inhibitor negative control sequence, for use as controls for non-sequence-specific effects in miRNA experiments. Mock-transfected controls did not produce any significant effect on any of the parameters analyzed.

### MicroRNA expression

#### Global miRNA expression

HCT 116 WT cells were seeded at 1×10^6^ cells/plate in 6 well plates in triplicate. After culture for 24 h, 25 µg/mL Cl-amidine was added into each well. Cells were harvested at 0, 12, and 24 h separately in RNase free EP tubes. Total RNA was extracted using TRIzol reagent (Ambion, Austin, TX). RNA concentration was determined by the Nanodrop 2000 (NanoDrop, Wilmington, DE). 100 ng of RNA from HCT 116 WT cells was used for the nCounter miRNA Expression Assay v1.2 (Nanostring Technologies, Seattle, WA) containing 800 miRNA's following the manufacturer's instructions.

#### miRNA-16 expression

Cells were seeded, exposed to Cl-amidine, and harvested at the same time points as described for global miRNA expression. For miRNA-16 detection, 10 ng of total RNA was used to reverse-transcribe to cDNA using TaqMan MicroRNA Reverse Transcription kit (Applied Bisystems, Foster City, CA) according to manufacturer's instructions and miRNA primers specific for hsa-miRNA-16 for detection and the small nuclear protein RNU6B (U6) for normalization (Applied Biosystems). qPCR measurement of miRNA-16 and U6 expression was performed using TaqMan miRNA Assays (Applied Bisystems) with the 7300 PCR Assay System (Applied Bioystems). The comparative threshold cycle (Ct) method was used to evaluate the relative abundance of miR-16 compared with U6 expression (fold changes relative to U6). All experimental treatments were carried out on three separate occasions; each time with three replicates.

### Western blot analysis

Western blots were carried out as described previously [30]. Antibodies used include anti-p53 (EMD Millipore, Rockland MA; Mouse Monoclonal, cat# op43, 1∶500), anti-p21^WAF1/Cip1^ (Sigma; Rabbit Polyclonal, cat# p1486, 1∶1000), anti-GAPDH (Cell Signaling, Rabbit Monoclonal, cat#2118, 1∶1000), and anti-PAD4 (Origene; Mouse Monoclonal, cat# TA504813, 1∶2000). Horseradish peroxidase-conjugated anti-mouse and anti-rabbit secondary antibodies were purchased from Amersham. Both secondary antibodies were diluted at 1∶2000. Western blot signal was detected by SuperSignal West Pico Chemiluminescent Substrate (Thermo Scientific, Rockford, IL) and developed onto Hyperfilm (GE Life Sciences, Piscataway, NJ).

### Cell cycle analysis

1×10^6^ cells were grow in 6 well plates in McCoy's 5A medium 1 day before treatment and then treated with 50 µg/mL Cl-amidine for corresponding times. The cells were harvested and fixed with ice cold ethanol. After washing with 1x Phosphate Buffered Saline (PBS) containing 0.5% Bovine Serum Albumin twice, the cells were incubated with DNA staining solution consisting of Propidium Iodide (PI; 10 µg/mL) and RNase (0.1 mg/mL) for 30 min at room temperature in the dark. The proportion of cells in each phase of the cell cycle was determined by DNA content stained with PI using a BD-LSR-II flow-cytometer (BD Biosciences, San Jose, CA). Data obtained were analyzed with BD FACSDiva Software (BD Biosciences). Each treatment was repeated on three separate occasions.

### mRNA analysis

Total RNA was extracted using Trizol reagent (Invitrogen, CA). One μg of total RNA served as template for single strand cDNA synthesis in a reaction using oligo(dT) primers and AMV reverse transcriptase (Promega Corp, WI) under conditions indicated by the manufacturer. PCR of cDNA samples was performed with samples amplified for 30 cycles of denaturation at 94°C for 30 s, annealing at 50°C for 30 s, and extension at 72°C for 30 s with final extension at 72°C for 10 min. The sequences for Real Time PCR primers used were: CCND1 Forward 5′-CCC TCG GTG TCC TAC TTC AA-3′, CCND1 Reverse 5′-AGG AAG CGG TCC AGG TAG TT-3′; CCND2 Forward 5′-TGG GGA AGT TGA AGT GGA AC-3′, CCND2 Reverse 5′-ATC CAC GTC TGT GTT GGT GA-3′; CCND3 Forward 5′-TGA TTT CCT GGC CTT CAT TC-3′, CCND3 Reverse 5′-AGC TTC GAT CTG CTC CTG AG-3′; CCNE1 Forward 5′-ATC CTC CAA AGT TGC ACC AG-3′, CCNE1 Reverse 5′-AGG GGA CTT AAA CGC CAC TT-3′; CDK6 Forward 5′-CCG TGG ATC TCT GGA GTG TT-3′, CDK6 Reverse 5′-TCT CCT GGG AGT CCA ATC AC-3′; GAPDH Forward 5′-GAG TCA ACG GAT TTG GTC GT-3′, GAPDH Reverse 5′-TTG ATT TTG GAG GGA TCT CG-3′ (Integrated DNA Technologies, Inc). Real-time PCR (qPCR) was performed using the 7300 Real-Time PCR Assay System (Applied Biosystems, CA) with Power SYBR green PCR master mix (Applied Biosystems, CA) and primers for CCND1, CCND2, CCND3, CCNE1, CDK6 and GAPDH according to the vendor's protocol. The CCND1, CCND2, CCND3, CCNE1, CDK6 gene expression was normalized by GAPDH gene expression. The fold change in the gene expression is relative to the vector treated (1x PBS) cells harvested at 24 h.

### Statistics

When more than two groups were compared, we determined statistical differences using a one-way analysis of variance, followed by a Scheffe's multiple comparison test. If two groups were compared, we used a Student's T-test. For global miRNA analysis, all data were imported into NSolver Analysis Software v1.0 (Nanostring Technologies) and normalized to the geometric mean of the 100 microRNAs with the highest expression values. Normalized data was imported into BRB-ArrayTools v4.1.0 for analysis. Prior to analysis, data were filtered where any value less than 10 was omitted and any microRNA missing in >50% of samples were excluded leaving 169 microRNAs for analysis. Class comparison test utilized Student's T-tests to compare microRNAs of treated vs. untreated cells. Trend tests used linear regression modeling on ordered categorical variables of 0 h, 12 h and 24 h. The Benjamini-Hochberg procedure was used to calculate false discovery rates. The P value chosen for significance in this study was 0.05.

## Results

### Cl-amidine causes a p53-dependent G1 arrest

To explore the functional implications of PAD inhibition, we first asked whether the PAD inhibitor, Cl-amidine, impacts the cell cycle. Using two independent p53 WT colon cancer cell lines, we found that Cl-amidine caused a cell cycle arrest at the G1 phase (Tables 1A and B; Figures S1A and S1B). Because the impact on the G1 phase was associated with an increase in p53 and p21 (Figure S2), we tested the hypothesis that Cl-amidine caused the G1 arrest through a p53-mediated mechanism. Results are consistent with this hypothesis. Unlike HCT 116 WT cells, Cl-amidine did not cause a G1 arrest in isogenic HCT 116 p53^−/−^ cells (Table 1C; Figure S1C). To confirm that this arrest was not due to off-target mechanisms, we knocked down PAD4 by siRNA (Figure S3A), then examined cell cycle changes. Consistent with the understanding that the G1 cell cycle arrest by Cl-amidine is due at least in part to its ability to suppress PAD activity, PAD4 knockdown also caused a G1 cell cycle arrest (Table 1D; Figure S3B) in HCT 116 cells. Cell cycle was examined 24 h after transfection.

**Table 1:** Percentage of cells in G1, S and G2 following exposure to Cl-amidine (50 µg/ml).

**A. HCT 116 Cells**			
**Time (h)**	**G1**	**S**	**G2**
0	49.6±7.5	34.7±11	14.4±2.9
2	55.8±0.7	25.6±4.3	15.2±2.8
8	58.8±6.4	22.7±5.5	17.4±3.7
24	78.8±5.3*	9.1±2.8**	11.4±4.1
**B. LS-180 Cells**			
**Time (h)**	**G1**	**S**	**G2**
0	62.3±1.8	18.0±1.1	16.7±4.3
2	67.1±0.8	16.1±1.27	12.5±0.4
8	67.5±5.0	14.9±1.1	14.3±4.2
24	68.7±0.3*	15.0±0.3	12.1±0.2
**C. HCT 116 p53^−/−^ Cells**			
**Time (h)**	**G1**	**S**	**G2**
0	50.7±8.3	25.8±7.8	18.4±0.5
2	47.1±0.8	28.6±1.5	19.0±1.3
8	44.4±4.6	28.1±0.8	19.1±0.5
24	51.2±11	22.8±4.3	18.5±0.5
**D. PAD4 siRNA HCT 116 Cells**			
**Treatment**	**G1**	**S**	**G2**
scrambled siRNA control	59.2±1.3	18.67±1.4	19.5±0.4
PAD4 siRNA	81.83±2.2*	6.4±0.9**	8±2.3***

* , indicates significant increase in the number of cells in G1 phase.

** , indicates significant decrease in the number of cells in S phase.

*** , indicates significant decrease in the number of cells in G2 phase.

### Cl-amidine causes a p53-dependent induction of miRNA-16

**Figure 1:**
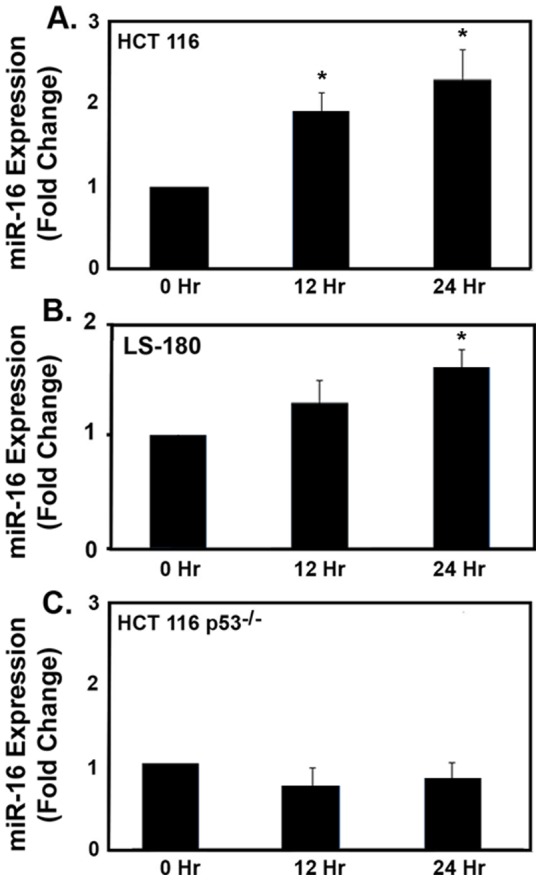
miRNA-16 expression changes in p53 WT colon cancer cells after exposure to Cl-amidine. (**A**) HCT 116 WT cells; (**C**) LS-180 cells.; (**C**) HCT 116 p53^−/−^ cells. Cells were exposed to 25 µg/mL Cl-amidine for indicated times (N = 9 plates per time point). Relative endogenous miR-16 expression levels were detected by qRT-PCR using Taqman primers and probes to detect mature miR-16 and the small nuclear RNA RNU6B (U6), an internal control. Relative miR-16 expression levels were normalized to the average value of the non-treated samples (0 h). *, indicates significant difference from the 0 hr control.

We have previously shown that the PAD inhibitor, Cl-amidine, stimulates apoptosis of inflammatory cells and modestly stimulates apoptosis in HT-29 colon cancer cells [4]. As well, we confirmed previous results from Li et al. [27] in that PAD4 knock-down and Cl-amidine treatment caused a G1 cell-cycle arrest in a p53-dependent manner (Table 1; Figures S1 and S2). Since miRNA's have emerged as key moderators of colonic cell proliferation and apoptosis [31], we examined global miRNA expression changes in HCT 116 colon cancer cells exposed to Cl-amidine. Table 2 shows there was a significant positive correlation in 11 miRNAs, and significant negative correlation in 7 miRNAs after exposure of HCT 116 cells to Cl-amidine for 0 h, 12 h, and 24 h. Based on the understanding that miRNA-16 is down-regulated in colon cancer [18], and miRNA-16 was shown to be the miRNA that was most up-regulated (significantly) by Cl-amidine (1.5 fold at 12 h; 2 fold at 24 h; Table 2), we decided to further explore the consequences and mechanisms of Cl-amidine-mediated miRNA-16 up-regulation. To confirm miRNA-16 up-regulation by Cl-amidine, we repeated the experiment and examined miRNA-16 activation by qRT-PCR. Consistent with the global miRNA analysis results, Figure 1A shows that there was increasing miRNA-16 expression with increasing time when HCT 116 cells were exposed to Cl-amidine, with significance being reached at both 12 h and 24 h (p<0.05). There was also an increase in miRNA-16 with increasing time exposed to Cl-amidine in the p53 WT LS-180 colon cancer cell line; significance being reached at 24 h (Figure 1B). The lower increase in miRNA-16 induction is consistent with the relatively modest G1 cell cycle arrest in the LS-180 cells (Table 1B; Figure S1B).

**Table 2:** miRNA expression changes with exposure to Cl-amidine (25 µg/ml).

	Correlation coefficient	Parametric p-value	FDR*	Unique id
1	0.949	0.0002625	0.0222	hsa-miRNA-16
2	−0.949	0.0002625	0.0222	hsa-miRNA-453
3	0.896	0.0029125	0.0984	hsa-miRNA-19b
4	0.896	0.0029125	0.0984	hsa-miRNA-31
5	−0.896	0.0029125	0.0984	hsa-miRNA-496
6	−0.843	0.0077506	0.218	hsa-miRNA-484
7	−0.926	0.0118115	0.285	hsa-miRNA-1283
8	0.791	0.0190648	0.293	hsa-miRNA-21
9	0.791	0.0190648	0.293	hsa-miRNA-221
10	0.791	0.0190648	0.293	hsa-miRNA-222
11	0.791	0.0190648	0.293	hsa-miRNA-331-3p
12	−0.819	0.0245418	0.322	hsa-miRNA-362-3p
13	0.849	0.0281225	0.322	hsa-miRNA-128
14	0.738	0.0323966	0.322	hsa-let-7a
15	0.738	0.0323966	0.322	hsa-miRNA-103
16	0.738	0.0323966	0.322	hsa-miRNA-200b
17	−0.738	0.0323966	0.322	hsa-miRNA-655
18	−0.756	0.0390752	0.365	hsa-miRNA-99a

* , indicates False Discovery Rate.

Because p53 enhances the post-transcriptional maturation of miRNA-16 [32], and Cl-amidine causes an induction in p53 (Figure S2), we examined whether the induction of miRNA-16 by Cl-amidine was p53-dependent. Figure 1C shows Cl-amidine was unable to cause an increase in miRNA-16 in HCT 116 p53^−/−^ cells, indicating miRNA-16 induction was indeed p53-dependent.

### The p53-dependent G1 arrest by Cl-amidine is regulated by miRNA-16

Given the observation that both the G1 arrest and increase in miRNA-16 by Cl-amidine were p53-dependent; that p53 enhances the maturation of miR-16 [32]; and that others have shown miRNA-16 causes a G1 arrest [13], [19], [20], [21], [22], [23], [24], [25], [26], we hypothesized that miRNA-16 is at the crossroads of the p53-dependent G1 arrest induced by PAD inhibition. To test this hypothesis, we knocked down miRNA-16 (Figure S3C) in HCT 116 cells, then exposed the cells to Cl-amidine. Results shown in Table 3A and Figure S3D indeed indicate that the G1 arrest caused by Cl-amidine in HCT 116 WT cells is dependent on miRNA-16, because in the absence of miRNA-16, Cl-amidine was unable to cause the cell cycle arrest. To confirm these results, we transfected HCT 116 cells with miRNA-16 siRNA and a miRNA-16 mimic. After incubation for 24 h with no transfection, transfection of miRNA-16 siRNA or the miR-16 mimic, cells were harvested and cell cycle examined. Table 3B shows the miRNA-16 mimic caused a G1 cell cycle arrest, supporting the hypothesis that the induction of miRNA-16 by Cl-amidine is a key event that leads to a G1 arrest in colon cancer cells with a p53 WT background. Taken together, we conclude that the p53-dependent G1 arrest is in turn dependent on the ability of PAD inhibition by Cl-amidine to up-regulate the expression of miRNA-16.

**Table 3:** Percentage of HCT 116 cells in G1, S and G2 following miRNA-16 knockdown and exposure to Cl-amidine (50 µg/ml) (A) or miRNA-16 mimic transfection (B).

**A. miRNA-16 siRNA + Cl-amidine (50 µg/mL)**			
**Time (h)**	**G1**	**S**	**G2**
0	64.6±0.96	13.6±0.70	15.9±1.10
2	64.0±0.83	13.6±0.51	16.4±0.46
8	63.6±0.97	11.0±0.76	15.4±0.46
24	60.4±0.56	16.5±0.12	17.0±0.91
**B. miRNA-16 mimic**			
**Treatment**	**G1**	**S**	**G2**
Control	63.9±0.10	15.6±0.76	17.5±0.81
miR16 siRNA	65.4±0.56	12.3±0.75	18.9±0.35
miR16 mimic	69.2±0.90*	9.8±1.00	17.7±0.80

* , indicates significant increase in the number of cells in G1 phase.

### Cl-amidine causes a downregulation of miRNA-16 targets involved in a G1 arrest

**Figure 2:**
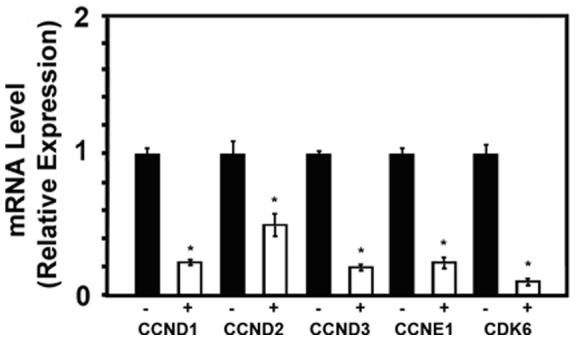
Cl-amidine suppresses mRNA levels of miRNA-16 targets. HCT 116 cells were treated with 1x PBS (−) or 25 µg/mL Cl-amidine (+) for 24 h., then RNA harvested for qPCR as described in methods. The CCND1, CCND2, CCND3, CCNE1, and cdk6 gene expression were normalized by GAPDH gene expression. *, indicates significant difference from the control (−) (p<0.05).

We have shown that miRNA-16 is at the crossroads of the G1 arrest in colon cancer cells by Cl-amidine. It would therefore stand to reason to examine the effects of Cl-amidine on miRNA-16 targets that are involved in a G1 cell cycle arrest. Such targets include: cyclin D1 (CCND1) [13], [19], [33], [34], [35], [36], cyclin D2 (CCND2)[13], cyclin D3 (CCND3) [33], cyclin E1 (CCNE1) [13], [21], [24], [35], and cyclin-dependent kinase-6 (cdk6) [35]. In examining the effects of Cl-amidine on these miRNA-16 targets, we found a significant decrease in all endpoints (Figure 2). This is consistent with the hypothesis that PAD inhibition by Cl-amidine activates miRNA-16, which in turn, down-regulates multiple genes known to be involved in G1 progression.

## Discussion

**Figure 3:**
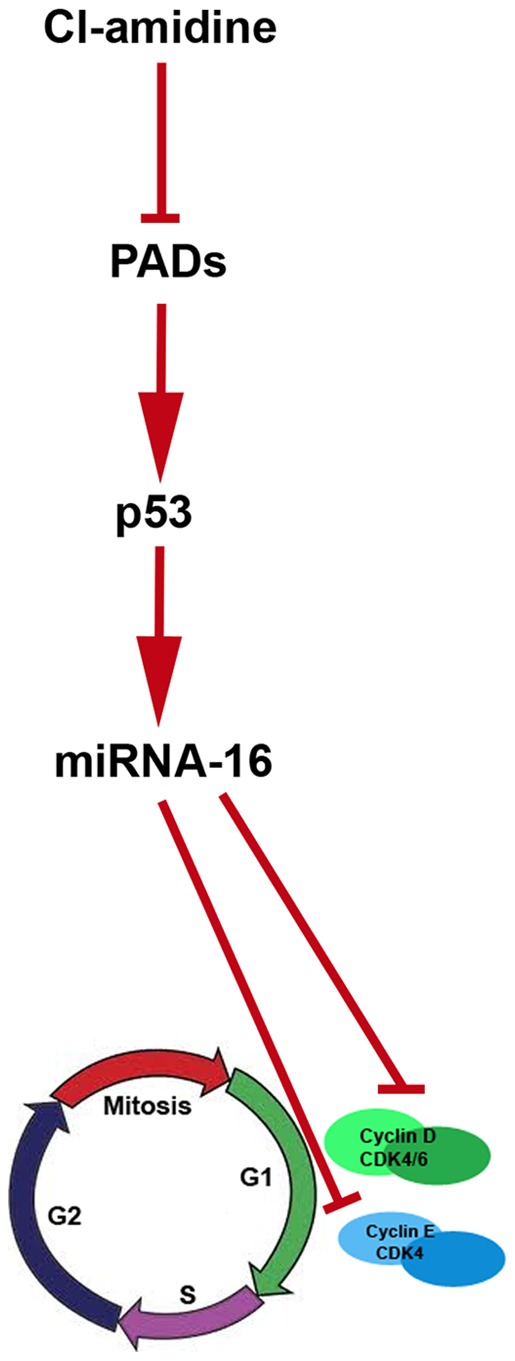
Model diagram generated by experiments carried out in this manuscript. The inhibition of PADs by Cl-amidine activates p53, which in turn activates miRNA-16. The activation of miRNA-16 causes a G1 cell cycle arrest, presumably by targeting cyclins D, E and/or cdk6.

PADs were first discovered in vertebrates in the early 1980's within the epidermis of newborn rats [37]. Since then, it has become increasingly apparent that PAD activity, with resultant protein citrullination, drives many inflammatory diseases and cancers, including colitis and colon cancer [1], [4], [5], [6]. Therefore, to develop therapies based on PAD signaling to suppress or prevent colon cancer, it is important to understand the mechanisms. We have identified that the inhibition of the PADs by our novel small molecule inhibitor (Cl-amidine) suppresses the high colon cancer risk disease, ulcerative colitis, in mice [4]. Here, we have extended these studies, and demonstrated that Cl-amidine causes a G1 cell cycle arrest in colon cancer cells, that this effect is mediated by up-regulating the expression of the putative colon cancer tumor suppressor microRNA, miRNA-16; and that this only occurs in the presence of the tumor suppressor protein, p53. Although we have yet to show that Cl-amidine suppresses colon cancer in mice, results here dissect the mechanisms by which PAD inhibition by Cl-amidine will likely suppress colon cancer. Given the close association between colon inflammation and colon cancer risk [38], [39], [40], [41], [42], our previous study showing that Cl-amidine suppresses colitis [4] also predicts that Cl-amidine can be used to prevent and/or treat colon cancer. In addition to suppressing inflammation, the present study indicates that an additional mechanism (for suppressing colon cancer by Cl-amidine) is through the p53 and miRNA-16 dependent G1 cell cycle arrest in colon cancer cells. The exact reasoning for the cell cycle arrest caused by the PAD inhibition-mediated increase in miRNA-16, however, remains to be elucidated. Although we saw an increase in p21^WAF1^ with Cl-amidine treatment (Figure S2), this does not appear to be the sole event that causes the G1 arrest, because of our miRNA-16 evidence (miRNA-16 ablation negates the G1 arrest by Cl-amidine: Table 3; Figures S3C and S3D). To this end, miRNA-16 has emerged as an especially key miRNA in the G1 cell cycle checkpoint. miRNA-16 targets several cell cycle regulators, including cyclin D1 (CCND1), cyclin D2 (CCND2), cyclin D3 (CCND3), cyclin E1 (CCNE1), cyclin-dependent kinase-1 (cdk1), cdk6, cdc25a, and ADP-ribosylation factor-like protein 2 (ARL2) [13], [19], [21], [24], [26], [33], [34], [35], [36], [43], [44]. Given their clear roles in driving the progression of dividing cells through the G1 phase, especially cdk6, cyclin D, and cyclin E, the ability of miRNA-16 to target such molecules is the most likely reason for our findings (Figures 2 and 3). Another separate set of experiments outside the scope of the current study involves the functional implications of other miRNA's shown to have differential expression following exposure of HCT 116 cells to Cl-amidine (Table 2).

Since Cl-amidine is a pan-PAD inhibitor [45], it remains to be shown which PAD is key to the induction of a G1 cell cycle arrest. Our finding that the down-regulation of PAD4 through siRNA causes a comparable G1 arrest to that of Cl-amidine (Tables 1A and 1D), indicates PAD4 is at least one of the key PAD enzymes responsible for cell cycle progression independent of the other PADs. Consistent with this finding is the understanding that PAD4 activity is elevated in colon adenocarcinomas [6]. However, due to the pan-PAD activity of Cl-amidine, we cannot rule out the impact of other PADs on cell cycle progression. This is especially true for PADs known to be expressed in cells of epithelial/carcinoma origin (those used in this study), including PAD1 [46], PAD2 [47] and PAD3 [7].

Our finding that Cl-amidine causes an induction of p53 (Figure S2) and inhibits cell growth in a p53-dependent manner is consistent with other groups [27], [48]. The mechanisms of p53 induction by PAD inhibition, however, remain unclear. PAD4 can regulate gene expression by catalyzing the posttranslational citrullination of a number of Arg residues in histone substrates [49], [50]. PAD4 can be recruited to histones by its interaction with p53 [27]. As a result, PAD4 gets recruited to the promoters of p53-targeted genes where it citrullinates histones and represses the transcription of p53-targeted genes. PAD4 can also citrullinate non-histone proteins that associate with p53 and are implicated in carcinogenesis. Notably, PAD4 can citrullinate Inhibitor of growth protein 4 (ING4), thereby inhibiting the ING4-mediated activation of p53 [51]. Such mechanisms support the finding that PAD inhibition by Cl-amidine causes p53 induction. Finally, we are exploring the possibility that p53 can be citrullinated, leading to the generation of SDS-insoluble aggregates, similar to a number of cancer associated p53 mutations [52]. If this is the case, the inhibition of p53 citrullination (by Cl-amidine) would lead to increased p53 levels by preventing this aggregation phenotype.

In conclusion, given the increasing understanding that PAD activity (and resultant citrullination) plays a key role in chronic diseases such as inflammatory diseases and cancer, it is important to better understand the mechanisms. Very little is currently known in this regard. With respect to cancer, here we have demonstrated that miRNA-16 plays a vital role in modulating the G1 cell cycle checkpoint induced by PAD inhibition in p53 WT colon cancer cell lines *in vitro*. Although the *in vivo* translation remains to be shown, these current experiments shed light into the mechanism by which PAD inhibition by our novel PAD inhibitor, Cl-amidine, works. Our mechanistic findings open up the possibility that PAD inhibitors alone, or in concert with p53 and/or miRNA-16 mimics, may have efficacy in the chemoprevention and/or treatment of colon cancer.

## Supporting Information

Figure S1 **Representative flow cytometric histograms on indicated colon cancer cell lines.** (A) Histograms at indicated time points showing Cl-amidine (50 µg/mL) induces a G1 phase arrest in HCT 116 cells, which are p53 WT. (B) Histograms at indicated time points showing Cl-amidine (50 µg/mL) induces a G1 phase arrest in LS-180 cells, which are p53 WT. (C) Histograms at indicated time points showing Cl-amidine (50 µg/mL) does not induce a G1 phase arrest in HCT 116 p53^−/−^ cells, which are deficient in p53.
(TIF)

Figure S2 **Cl-amidine causes an induction in p53 and p21 in HCT 116 cells, but not in HCT 116 p53^−/−^ cells.** Cells were exposed to indicated concentrations of Cl-amidine for 24 h, then harvested for western blot analysis. Numbers under the bands are GAPDH-adjusted densitometry values relative to the control group (0 µg/mL Cl-amidine).
(TIF)

Figure S3 (**A, B**)**. Knocking down PAD4** (**A**) **causes a G1 cell cycle arrest in HCT 116 cells** (**B**)**.** (A) Western blot analysis showing PAD4 knockdown with siRNA to PAD4. Numbers under the bands are GAPDH-adjusted densitometry values relative to the scrambled siRNA control group. (B) Representative cell cycle plots after 24 h. transfection with either scrambled siRNA or siRNA to PAD4. (**C, D**)**.** After knocking down miR-16 (C), Cl-amidine (50 µg/mL) does not cause a G1 cell cycle arrest in HCT 116 cells (D). (C) Quantification of miRNA-16 expression by Q-PCR. *, indicates significant reduction in miRNA-16 expression at 24 h post-transfection compared with scrambled control siRNA. Note that Cl-amidine did not affect the efficiency of miRNA-16 knockdown. (D) Representative flow cytometric histogrtams after 24 h. transfection with anti-miR-16, then exposure to Cl-amidine (50 µg/mL) for indicated time points.
(TIF)
