# Supplementary figures and images for: The Induction of microRNA-16 in Colon Cancer Cells by Protein Arginine Deiminase Inhibition Causes a p53-Dependent Cell Cycle Arrest
Source: PLoS One. 2013 Jan 7;8(1):e53791. doi: 10.1371/journal.pone.0053791 (PMC3538596; doi:10.1371/journal.pone.0053791)

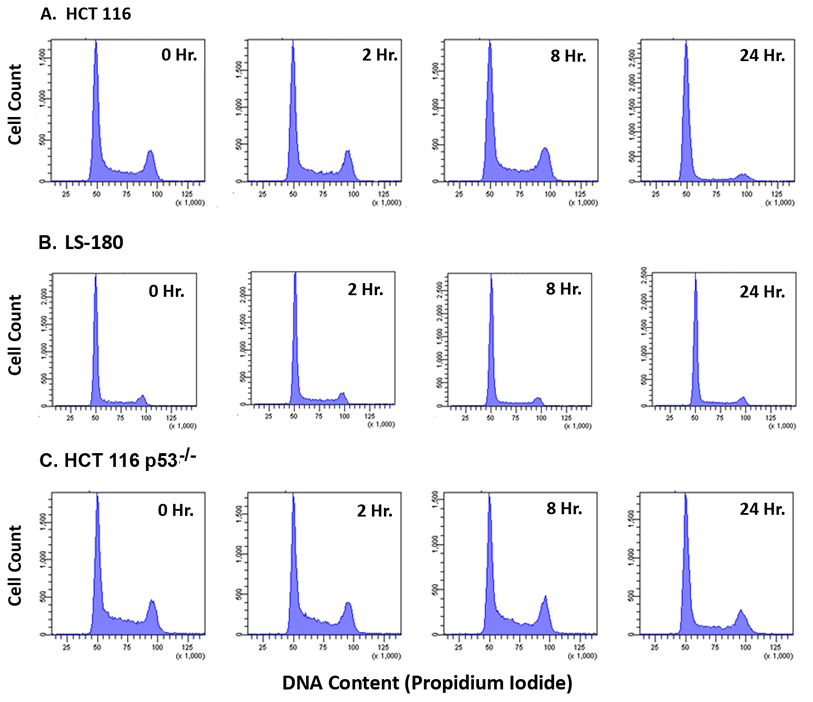

Supplement: Figure S1 — Representative flow cytometric histograms on indicated colon cancer cell lines. (A) Histograms at indicated time points showing Cl-amidine (50 µg/mL) induces a G1 phase arrest in HCT 116 cells, which are p53 WT. (B) Histograms at indicated time points showing Cl-amidine (50 µg/mL) induces a G1 phase arrest in LS-180 cells, which are p53 WT. (C) Histograms at indicated time points showing Cl-amidine (50 µg/mL) does not induce a G1 phase arrest in HCT 116 p53−/− cells, which are deficient in p53. (TIF) [file pone.0053791.s001.tif]

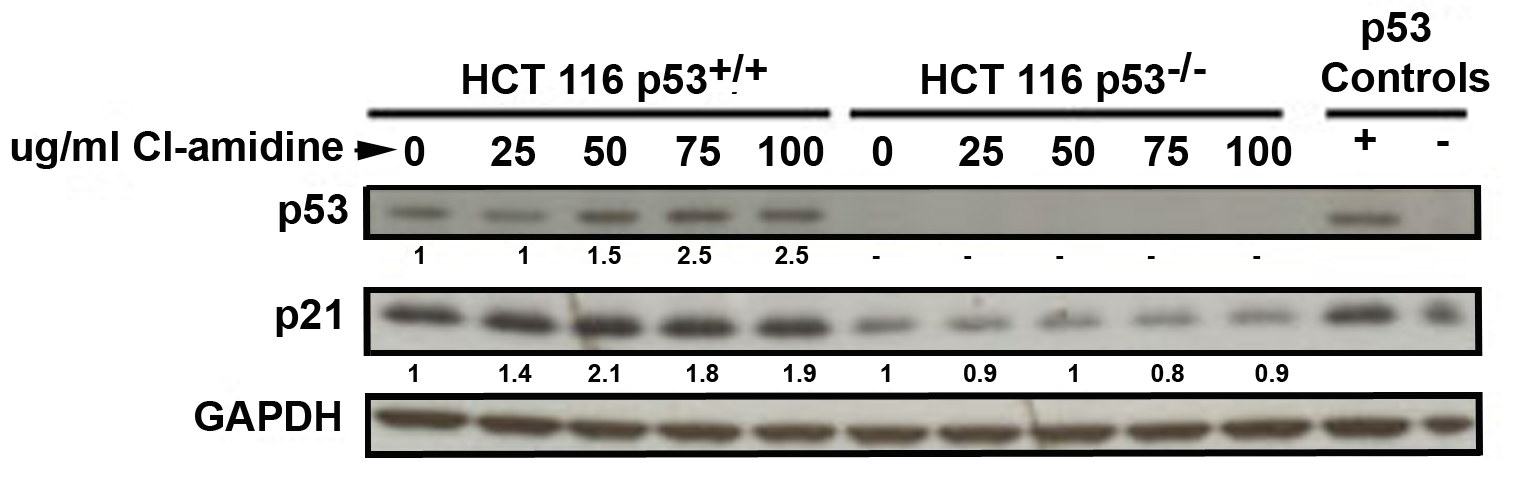

Supplement: Figure S2 — Cl-amidine causes an induction in p53 and p21 in HCT 116 cells, but not in HCT 116 p53−/− cells. Cells were exposed to indicated concentrations of Cl-amidine for 24 h, then harvested for western blot analysis. Numbers under the bands are GAPDH-adjusted densitometry values relative to the control group (0 µg/mL Cl-amidine). (TIF) [file pone.0053791.s002.tif]

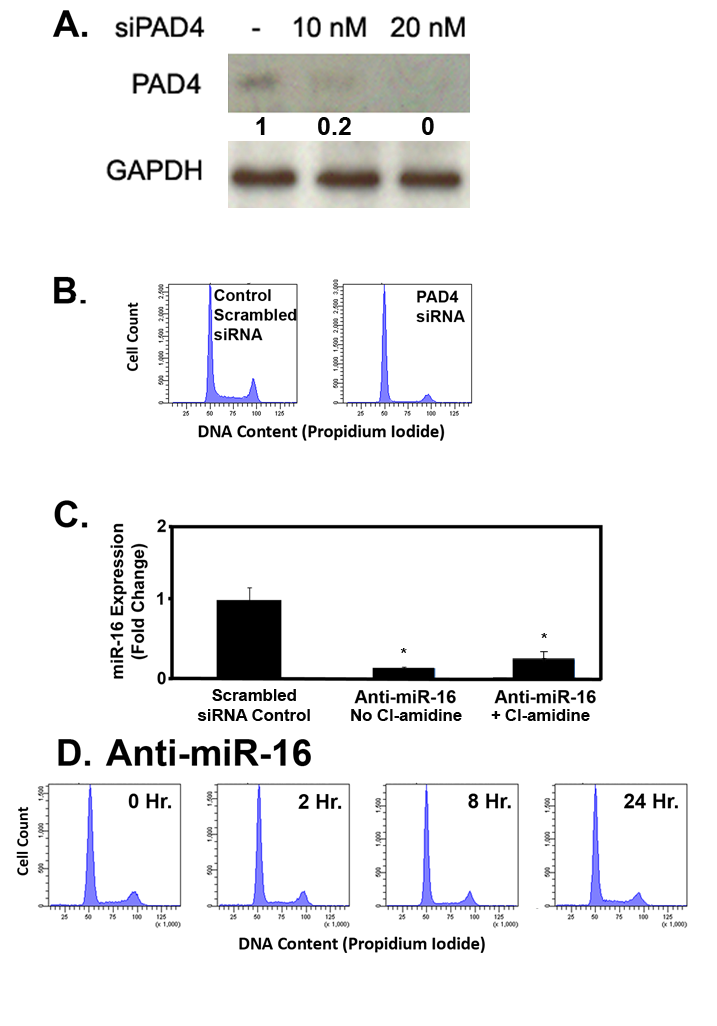

Supplement: Figure S3 — (A, B). Knocking down PAD4 (A) causes a G1 cell cycle arrest in HCT 116 cells (B). (A) Western blot analysis showing PAD4 knockdown with siRNA to PAD4. Numbers under the bands are GAPDH-adjusted densitometry values relative to the scrambled siRNA control group. (B) Representative cell cycle plots after 24 h. transfection with either scrambled siRNA or siRNA to PAD4. (C, D). After knocking down miR-16 (C), Cl-amidine (50 µg/mL) does not cause a G1 cell cycle arrest in HCT 116 cells (D). (C) Quantification of miRNA-16 expression by Q-PCR. *, indicates significant reduction in miRNA-16 expression at 24 h post-transfection compared with scrambled control siRNA. Note that Cl-amidine did not affect the efficiency of miRNA-16 knockdown. (D) Representative flow cytometric histogrtams after 24 h. transfection with anti-miR-16, then exposure to Cl-amidine (50 µg/mL) for indicated time points. (TIF) [file pone.0053791.s003.tif]
